# Golgi Cisternal Unstacking Stimulates COPI Vesicle Budding and Protein Transport

**DOI:** 10.1371/journal.pone.0001647

**Published:** 2008-02-20

**Authors:** Yanzhuang Wang, Jen-Hsuan Wei, Blaine Bisel, Danming Tang, Joachim Seemann

**Affiliations:** 1 Department of Cell Biology, University of Texas Southwestern Medical Center, Dallas, Texas, United States of America; 2 Department of Molecular, Cellular and Developmental Biology, University of Michigan, Ann Arbor, Michigan, United States of America; University of Geveva, Switzerland

## Abstract

The Golgi apparatus in mammalian cells is composed of flattened cisternae that are densely packed to form stacks. We have used the Golgi stacking protein GRASP65 as a tool to modify the stacking state of Golgi cisternae. We established an assay to measure protein transport to the cell surface in post-mitotic cells in which the Golgi was unstacked. Cells with an unstacked Golgi showed a higher transport rate compared to cells with stacked Golgi membranes. Vesicle budding from unstacked cisternae *in vitro* was significantly increased compared to stacked membranes. These results suggest that Golgi cisternal stacking can directly regulate vesicle formation and thus the rate of protein transport through the Golgi. The results further suggest that at the onset of mitosis, unstacking of cisternae allows extensive and rapid vesiculation of the Golgi in preparation for its subsequent partitioning.

## Introduction

Proteins and lipids are exchanged between Golgi cisternae by transient tubular connections and vesicles that form at the rims of one cisterna and fuse with the next in the secretory pathway [1]–[3]. Trafficking through the Golgi may be mediated by cisternal maturation, or vesicular transport [4]–[6]. The maturation model proposes that cargo is transported by modification of the cisternae, while Golgi enzymes are recycled via retrograde transport of COPI vesicles. In the vesicular transport model, Golgi cisternae remain stable and cargo is transported through them by COPI vesicles. In both cases, the budding rate of vesicles determines the rate of transport across the Golgi [7]. In the vesicular transport model, vesicles carry cargo while in the maturation model, vesicles are essential to maintain the correct location of Golgi resident proteins.

During intra-Golgi transport, COPI vesicles are tethered by a protein complex comprised of GM130, p115 and giantin. Tethering factors aid the assembly of the SNARE complexes and establish the initial contact between the vesicle and the target membrane [8]–[10]. p115 tethers membranes by binding to giantin on COPI vesicles and GM130 on the Golgi. Because p115 can link two membranes together, it initiates stacking of cisternae in post-mitotic cells by bridging GM130 and giantin on opposite cisternae. Once stacks are formed, the link between the cisternae is strengthened by the stacking protein GRASP65 [11]. GRASP65 is a peripheral Golgi protein that forms homodimers, which further oligomerize to hold adjacent cisternae together [12]. The interaction between GM130 and GRASP65 indicates that cisternal stacking and vesicle transport might be linked [13]. Whether stacking directly regulates cargo transport through the Golgi is so far untested.

The function of stacking is still unclear, but it may function as a “flux regulator” – regulating the flow of cargo through the secretory pathway. It has been suggested that stacking improves the efficiency of vesicular transport between the cisternae [11]. The close arrangement of cisternae ensures the movement of the vesicles from one cisterna to another in the most efficient manner. An extension of this model suggests that transport through the stack depends on the rate at which COPI vesicles bud and fuse. With stacked cisternae, only the rims are accessible for budding and fusion, but as cisternae unstack, more membrane would become available so that the flux of material through the stack could increase.

Changes in the organization of the Golgi are particularly apparent during cell division, during which it disassembles and then reforms in the daughter cells [14], [15]. The disassembly is at least partially caused by the inhibition of vesicle fusion [16]. Phosphorylation of GM130 on serine 25 by cdk1/cyclinB1 inhibits the assembly of the GM130-p115-giantin tether and thus the fusion of COPI vesicles [17]. Continuous vesicle formation without fusion during mitosis leads to an accumulation of vesicles and thus fragmentation of the Golgi [15], [18]. Mitotic disassembly of the Golgi also involves unstacking. Phosphorylation of GRASP65 breaks GRASP65 oligomers and leads to unstacking of the cisternae [12]. So far it is unclear whether unstacking affects vesicle-driven Golgi disassembly at the onset of M-phase.

## Results

### GRASP65 mediates stacking of Golgi cisternae in post-mitotic cells

**Figure 1:**
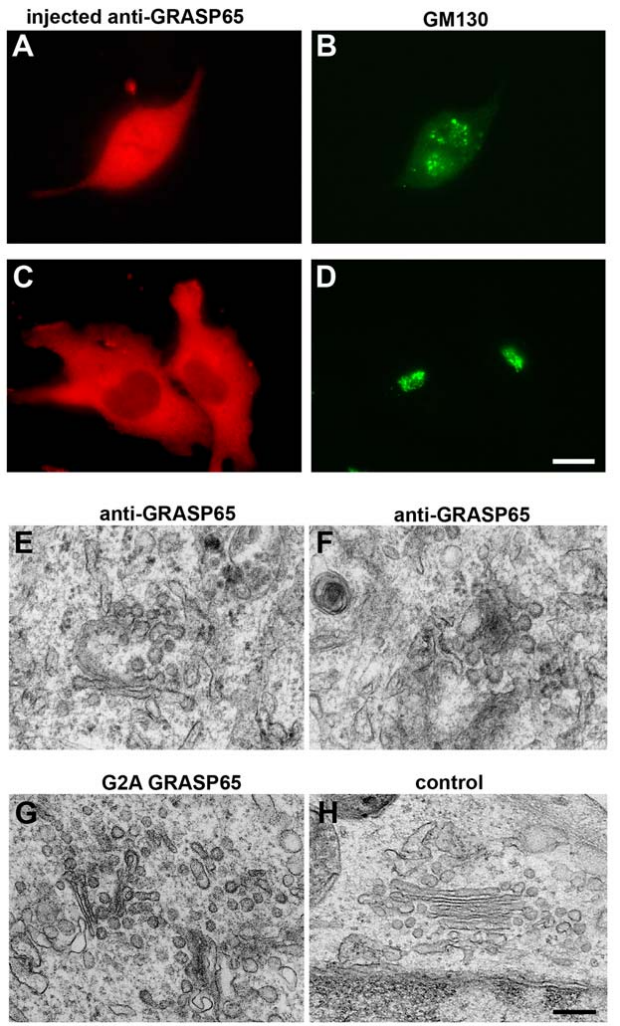
Microinjection of antibodies to GRASP65 or recombinant G2A GRASP65 prevents post-mitotic Golgi stack formation. (A–D) NRK cells in metaphase were microinjected with affinity-purified antibodies to GRASP65. The cells were fixed either immediately (A, B) or after cell division (C, D). The injected antibodies (A, C) and the Golgi marker GM130 (B, D) were stained. The microinjected cells divided into two daughter cells, each of which has a reformed Golgi ribbon. Bar, 15 µm. (E–H) NRK cells in metaphase were microinjected with anti-GRASP65 (E, F), G2A GRASP65 protein (G), or non-specific rabbit IgGs as a control (H). FITC-labeled dextran was co-injected as a marker. Once cell division was complete, all non-injected cells were removed and the remaining injected cells were processed for EM. Shown are EM micrographs of the reassembled Golgi in the injected cells. Note that in control IgG injected cells, the Golgi stacks are properly reassembled. In cells injected with either anti-GRASP65 or G2A GRASP65, however, cisternae appear disorganized and poorly aligned. In close proximity to the cisternae, there are a large number of vesicles and budding profiles, which appear to be coated. Bar, 0.5 µm.

To explore the role of Golgi stacking in protein trafficking, we used GRASP65 as a tool to modify the stacking state of the Golgi. We took advantage of the naturally occurring unstacking during mitosis. Mitotic NRK cells were microinjected with GRASP65 antibodies, non-myristoylated (G2A mutant) GRASP65 protein, or rabbit IgGs as a control. Injected cells were identified by staining the injected antibodies with secondary antibodies. Fig. 1 A shows a representative cell in prometaphase that was fixed immediately after injection. Staining for GM130 shows the typical pattern of mitotic Golgi fragments in close proximity to the two spindle poles [19] (Fig. 1 B). After 45 min, the cells divided and a Golgi reformed in both daughter cells (Fig. 1 C, D), suggesting that the injected proteins had no effect on mitotic progression. In addition, inhibition of GRASP65 function with either antibodies or mutant proteins interfered with Golgi stack formation but not Golgi ribbon formation (Fig. 1D–G), consistent with previous reports [12], [13], [20].

We further analyzed the Golgi morphology in injected cells using high resolution EM. The non-injected cells were scraped off and the injected cells (identified by the co-injected fluorescent marker) were analyzed by EM. In control-injected cells, a typical Golgi reformed with closely aligned, flattened cisternae (Fig. 1 H). In contrast, injection of G2A GRASP65 (Fig. 1 G) or GRASP65 antibodies (Fig. 1 E, F) severely interrupted the reformation of Golgi stacks. Some cisternae were arranged in close proximity to each other, but the majority were not properly aligned or stacked. This suggests that unstacked Golgi membranes can be obtained using GRASP65 antibodies or the G2A mutant protein to inhibit GRASP65 function. We also observed an increase of vesicular structures close to the cisternae. The vesicles were uniform in size (about 70 nm in diameter) and often coated. In addition, budding profiles at the cisternal membranes were often observed, indicating that the unstacked Golgi was capable of forming vesicles.

### Transport to the plasma membrane is accelerated when the Golgi is unstacked

In principle, the accumulation of vesicles could be caused either by increased vesicle budding, which stimulates protein delivery to the cell surface, or by inhibition of vesicle fusion, which slows down cargo transport through the Golgi. To distinguish between these two possibilities, we examined the kinetics of CD8 transport, a plasma membrane protein normally not expressed in this type of cells [19]. We established an assay to monitor the appearance of CD8 on the cell surface by recording the recruitment of a fluorescence-labeled CD8 antibody from the medium to the plasma membrane by time-lapse microscopy. The change in fluorescence intensity per time unit indicates the speed of transport and is independent of the starting point of the measurement. An advantage of this assay is that secretion can be followed in individual cells independent of expression levels and the starting times of protein transport, as in post-mitotic cells.

**Figure 2:**
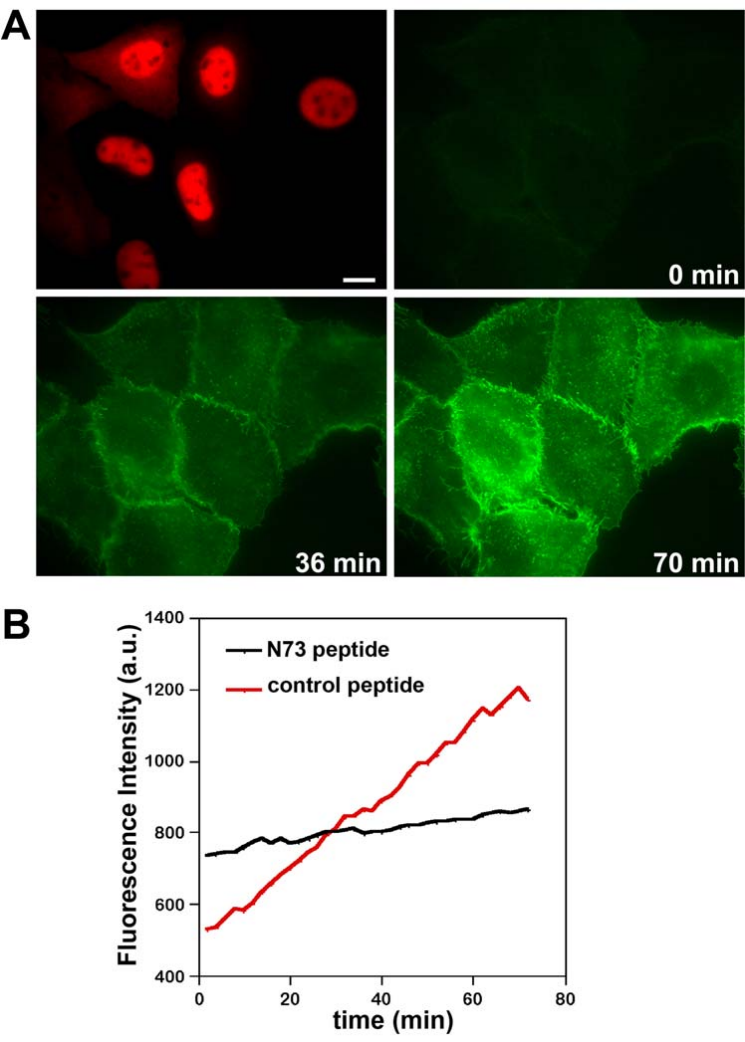
CD8 transport assay. (A) A plasmid encoding the plasma membrane protein CD8 was microinjected together with Texas-Red dextran (top left panel, red) into NRK cells. After 45 min, a fluorescently labeled antibody against the luminal domain of CD8 was added to the medium. The recruitment of the antibody onto the cell surface was analyzed by time-lapse microscopy in 2 min intervals over 70 min (Supplemental Movie S1). The fluorescence intensity on the cell surface increased over time, representing the arrival of CD8 from the Golgi to the plasma membrane. Bar, 15 µm. (B) Quantitation. To validate the transport assay, a peptide (N73) that inhibits intra-Golgi transport, or a control peptide (wt), were co-injected into NRK cells along with the CD8 plasmid and Texas-Red dextran and assayed for CD8 transport as in (A). The mean fluorescence intensity of the cells in each frame was quantified and plotted (red curve: control, n = 10; black curve: N73 peptide, n = 6); it decreased from 9.7 per min for control-injected cells to 2.4 per min for N73-peptide injected cells. CD8 transport was therefore inhibited by 75%.

This assay was validated by microinjection of a GM130 N-terminal peptide (N73), a known inhibitor of Golgi transport that disrupts the GM130-p115 tethering complex [21]. This peptide leads to an accumulation of COPI vesicles as well as a 65% reduction of protein transport to the cell surface [22]. Here we injected the N73 peptide or a control peptide together with the CD8 plasmid (Fig. 2 A). The N73 peptide displaced p115 from the Golgi, which is consistent with previous results [21], [22], but the control peptide had no effect (not shown). Injected cells were identified after 45 min by the injected fluorescent dextran, and recruitment of the fluorescent CD8 antibody to the plasma membrane was monitored at 2 min intervals over 70 min (Fig. 2 A, Movie S1). The increase in fluorescence intensity (Fig. 2 B) per minute was reduced from 9.7 (control) to 2.4 (N73 peptide), which corresponds to a 75% inhibition of CD8 transport. This is similar to the previously reported 65% inhibition in fixed cells where the viral plasma membrane protein VSV-G was used as cargo [22].

**Figure 3:**
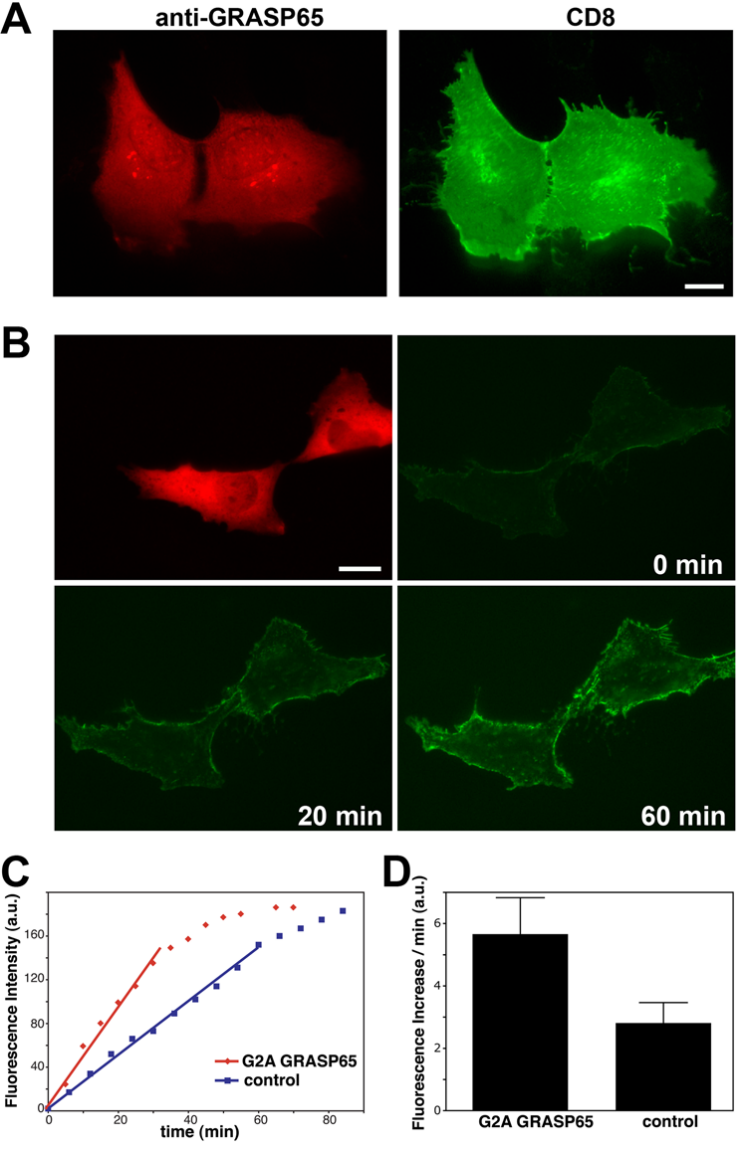
CD8 transport to the plasma membrane is accelerated through unstacked Golgi membranes. (A) Metaphase NRK cells were microinjected with affinity-purified antibodies against GRASP65 together with a plasmid encoding the plasma membrane protein CD8. After cell division, CD8 was expressed in the daughter cells and transported to the plasma membrane. 45 min before fixation, cycloheximide was added to inhibit protein translation. Cells were double labeled with a monoclonal antibody against CD8 and the microinjected polyclonal antibodies against GRASP65. Note that CD8 was transported to the cell surface in cells where Golgi stack formation was inhibited by the injected GRASP65 antibodies. Bar, 15 µm. (B) Metaphase NRK cells were injected with G2A GRASP65 protein together with a CD8 plasmid and Texas-Red dextran as a marker. At the end of mitosis, a fluorescently labeled antibody against CD8 was added to the medium. The fluorescence increase was followed by time-lapse microscopy in 2 min intervals over 70 min (Movie S2). Bar, 15 µm. (C) Quantitation. The mean fluorescence intensity of the cells in each frame was quantified and plotted (red curve: G2A GRASP65 injection, blue curve: control injection). The data shown were obtained from one pair of divided cells for each condition. Note the increased rate of transport in cells injected with G2A GRASP65. (D) The transport rate of CD8 to the plasma membrane increased two-fold in cells with unstacked Golgi cisternae. Quantitation from three independent experiments (mean±SD; p<0.05). Appearance of CD8 per minute at the cell surface was stimulated by 90% in G2A GRASP65 injected cells (n = 34) compared to control cells (n = 44).

To test whether the unstacked Golgi was capable of transporting proteins, prometaphase cells were injected with GRASP65 antibodies together with a plasmid encoding CD8. Two hours later, immunofluorescence analysis showed that CD8 was transported to the cell surface in both daughter cells, as shown by a typical staining pattern for plasma membrane proteins (Fig. 3 A). This showed that unstacked Golgi membranes were still transporting cargo proteins. We then measured the rate of transport in post-mitotic cells with disrupted Golgi stacks. Unsynchronized NRK cells in metaphase were injected with G2A GRASP65 protein to inhibit stacking, together with the CD8 plasmid and Texas Red dextran as a marker. After mitosis, CD8 was synthesized and its arrival on the plasma membrane was monitored by time-lapse microscopy (Fig. 3 B, Movie S2). The mean fluorescence of a representative cell pair from each condition showed that the CD8 signal increased linearly within the first 30 min and that CD8 accumulated faster on the plasma membrane in cells with an unstacked Golgi (Fig. 3 C). The results from three independent experiments showed that transport is nearly doubled in cells injected with G2A GRASP65 compared to control cells (Fig. 3 D). The signal was increased to 5.7±0.9 fluorescence units/min compared to 3.0±0.5, which corresponds to an increase of 90% in CD8 transport. This indicates that unstacking of the Golgi increases the transport rate to the cell surface, perhaps by accelerating vesicle budding (Fig. 1 E–G).

### Unstacked Golgi cisternae generate vesicles more efficiently

**Figure 4:**
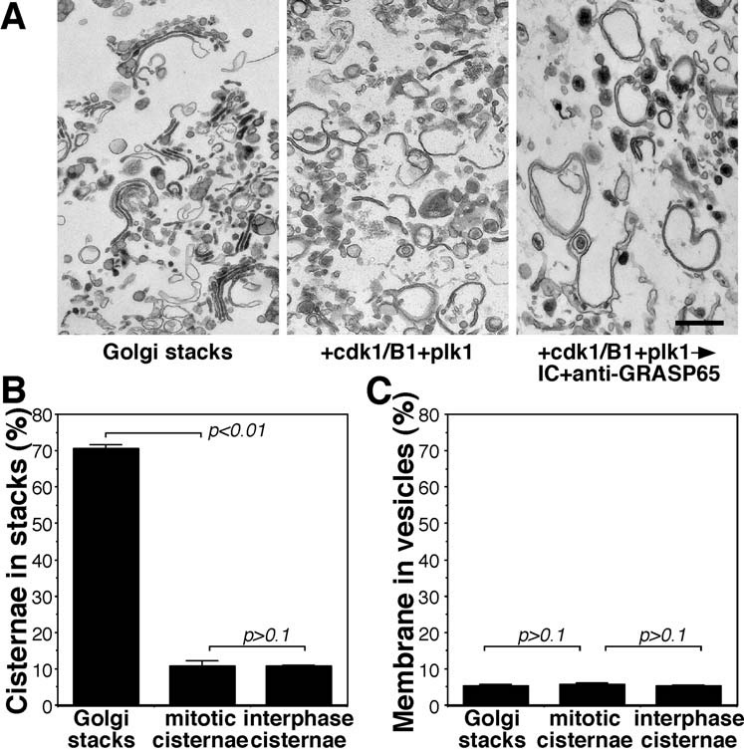
Inhibition of stacking by antibodies against GRASP65. (A) Rat liver Golgi membranes (RLG) were incubated with cdk1/cyclin B1 and plk1 and fixed either directly or after further incubation with interphase HeLa cell cytosol in the presence of GRASP65 antibodies. Representative EM micrographs of the membranes are shown in (A). Incubation with mitotic kinases led to unstacking of Golgi cisternae (cdk1/cyclin B1+plk1), and the subsequent reformation of stacks upon treatment with interphase cytosol was inhibited by antibodies against GRASP65. Bar, 0.5 µm. (B, C) Quantitation of (A) by the intersection method (mean±SD). Upon treatment with mitotic kinases, membranes in stacks were reduced to 11%, compared to 71% for untreated Golgi membranes. When interphase single cisternae were generated in the presence of GRASP65 antibodies and interphase cytosol, the percentage of membranes in stacks remained unchanged (B). During this process, the single cisternal membranes remained intact and did not vesiculate, as the percentage of membranes in vesicles was unchanged between all groups (C). Statistical significance was assessed by a two-tailed Student's *t* test.

We then used an *in vitro* vesicle budding assay to determine whether unstacked single cisternae can form COPI vesicles more efficiently than stacks. We treated Golgi membranes with the mitotic kinases cdk1/cyclinB1 and plk1 to phosphorylate GRASP65 and thus unstack the Golgi. Dephosphorylation with interphase cytosol in the presence of GRASP65 antibodies inhibited GRASP65 oligomerization [12] and thus restacking, resulting in single interphase cisternae (Fig. 4 A). EM analysis showed that 71% of the cisternae were in stacks (Fig. 4 B). After incubation with cdk1/cyclinB1 and plk1, the majority of the cisternae were separated from each other and the percentage of stacked cisternae dropped to 11%. When further treated with interphase cytosol and GRASP65 antibodies, the percentage of cisternae in stacks remained unchanged at 11% (Fig. 4 B). The percentage of membranes in vesicles remained unchanged at 5% throughout each treatment, showing that the decrease in stacked cisternae was caused by unstacking, rather than by a conversion of the membranes into vesicles (Fig. 4 C).

**Figure 5:**
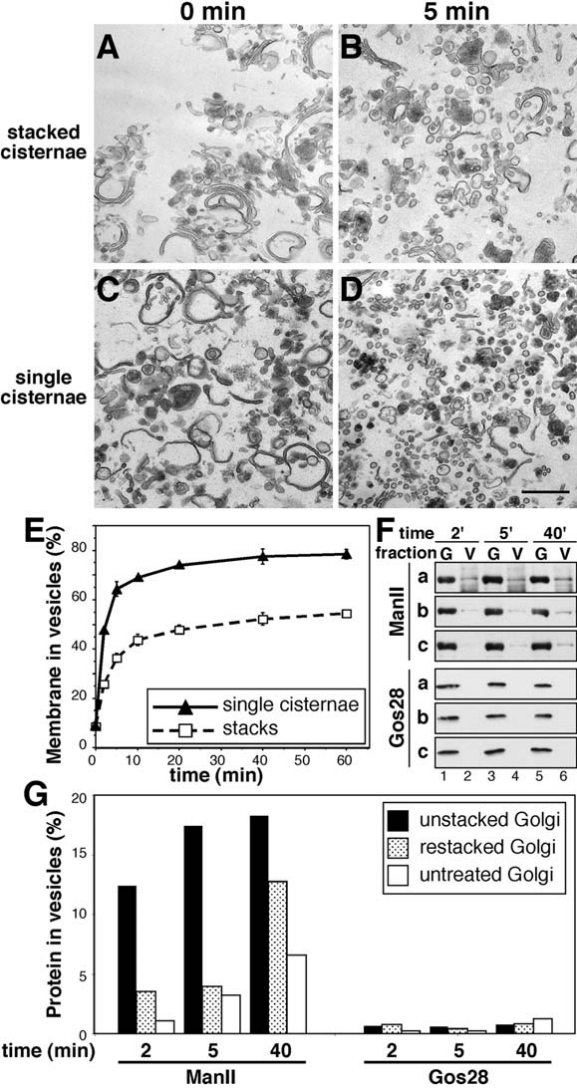
Unstacking of Golgi cisternal membranes increases vesicle generation. (A–D) EM micrographs of untreated Golgi membranes (A), or interphase single cisternae (C) generated by sequential treatment with cdk1/cyclin B1 and plk1 and interphase cytosol in the presence of GRASP65 antibodies. Membranes in (A) and (C) were treated with coatomer and ARF1 to allow vesicles to form. Membranes were fixed at different time points and processed for EM. Shown in (B) and (D) are membrane profiles after 5 min incubation. Note that budding from single cisternae was increased compared to that from stacked membranes. Within 5 min, nearly all single cisternae were converted into vesicles (D), while budding from stacked cisternal membranes was less efficient (B). Bar, 0.5 µm. (E) Quantitation of (A–D). Untreated Golgi membranes as in A, or single cisternae as in C were incubated with coatomer and ARF1 and then analyzed by EM. Shown is the quantitation of EM micrographs by the intersection method. The rate of vesicle formation from single cisternae was doubled (t_1/2_ = 1.6 min) compared to stacked membranes (t_1/2_ = 3.5 min). 78.5% of the membranes from single cisternae were converted into vesicles with a half-time of 1.6 min. The formation of the same amount of vesicles from stacked membranes took 12.4 min. Statistical significance assessed by a two-tailed Student's *t* test showed that p<0.01 for all time points except 0 min. (F) Untreated Golgi membranes (c, Golgi stacks) were treated with cdk1/cyclin B1 and plk1 followed by further incubation with interphase cytosol in the presence (a, unstacked cisternae) or absence (b, restacked cisternae) of GRASP65 antibodies. These membranes were incubated with coatomer and ARF1 for the indicated times followed by separation of the Golgi membranes (G) and vesicles (V) by centrifugation. Equal fractions of the samples were analyzed by Western blotting for ManII and Gos28. (G) Quantitation of (F). ManII was recruited more efficiently into the vesicle fractions from single cisternae compared to stacks or restacked membranes, whereas the t-SNARE Gos28 remained in the Golgi fraction. Results represent 3 independent experiments.

We then tested the effect of Golgi unstacking on the rate of vesicle formation. Single cisternae or stacks were incubated with ARF1 and coatomer to allow budding. EM analysis showed that within 5 min, almost all of the single cisternae were converted into vesicles (Fig. 5 D). This contrasts with stacked membranes, where vesicles formed to a much lesser extent and stacks were still prominent (Fig. 5 B). Within 5 min, 64% of the single cisternae were converted into vesicles, whereas only 36% of the stacks were vesiculated (Fig. 5 E). After 60 min, 78.5% of the single cisternae were converted into vesicles compared to 54.5% from stacks. This result shows that vesicle formation increased when the Golgi membranes were unstacked. Furthermore, the high degree of vesiculation seen with unstacked membranes within a short time is comparable to Golgi breakdown during mitosis, whereas stacks never reach similar levels. Single cisternae vesiculated with a half-time of 1.6 min compared to 3.5 min from stacks, suggesting that budding of COPI vesicles increased two-fold. The increase of vesicle formation from unstacked cisternae was also confirmed by differential sedimentation to separate vesicles from Golgi remnants followed by Western blotting analysis (Fig. 5 F). The Golgi resident enzyme α-mannosidase II, which has been shown to be incorporated into COPI transport vesicles [23], shifted more efficiently into the vesicle fraction from single cisternae compared to restacked or untreated Golgi membranes (Fig. 5 F, G). This is in contrast to the Golgi localized t-SNARE Gos28, which essentially remained in the Golgi membranes. Taken together, these results show that vesicles form more efficiently from unstacked Golgi membranes, resulting in a stimulation of transport to the cell surface. This suggests that stacking of cisternae slows down the rate of vesicle formation, perhaps by reducing the membrane surface area available for vesicle budding to the rims of the stacks. In contrast, vesicles may bud from the entire surface of single cisternae, which explains the higher rate of vesicle formation.

## Discussion

In this study we investigated the biological role of Golgi cisternal stacking in the regulation of protein transport through the Golgi. The data presented here indicate that vesicles form more efficiently from unstacked cisternae, leading to an increased rate of protein transport through the Golgi apparatus to the cell surface. Previous work has shown that stacking of Golgi cisternae is mediated by GRASP65 oligomers that link adjacent cisternae together into stacks [12], [13]. Mitotic phosphorylation of GRASP65 by recombinant cdk1/cyclinB1 and plk1 breaks the oligomers, inducing unstacking of the membranes [12], [24]. Upon dephosphorylation, GRASP65 oligomers reform, leading to the reformation of stacks [12], [25]. Studies using RNAi in Drosophila cells showed that knockdown of dGRASP (the sole GRASP protein in Drosophila) resulted in disassembly, of the Golgi stacks, at least partially, into single cisternae and vesicles [26]. In mammalian cells, microinjections of GRASP65 antibodies or a mutant protein into mitotic cells, in which the Golgi is disassembled, inhibited post-mitotic Golgi stack formation [12]; knockdown of GRASP65 reduced the average number of cisternae per stack from 6 to 3 [27], although a different report using the same RNA oligos showed that Golgi ribbon formation rather than stack formation was affected [28]. GRASP65 forms mitotically regulated oligomers, and the expression of a GRASP mutant that can not be de-oligomerized inhibited mitotic Golgi disassembly [20]. Finally, expression of a caspase-resistant form of GRASP65, which is cleaved during apoptosis, partially prevented Golgi fragmentation in apoptotic cells [29]. Taken together, these results provide strong evidence that GRASP65 is essential for Golgi stacking. Here we provide additional evidence and show that the inhibition of GRASP65 leads to Golgi membrane unstacking *in vitro* as well as in tissue culture cells. This also allowed us to use GRASP65 as a tool to modify the stacking state of the Golgi and thus determine the subsequent effect on protein trafficking.

We created single cisternae by adding recombinant GRASP65 or antibodies against GRASP65 to block stack reformation when GRASP65 was dephosphorylated. When these membranes were assayed for COPI vesicle budding, we found that the rate of vesicle formation was increased compared to stacked Golgi membranes. This suggests that stacking of cisternae slows down the rate of vesicle formation, perhaps by reducing the membrane surface area available for vesicle budding at the rims of the stacks. In contrast, vesicles may bud from the entire surface of single cisternae, which would explain the higher rate of vesicle formation.

Consistent with the increased rate of vesicle formation *in vitro*, our experiments in intact cells confirmed that stacking reduced trafficking from the Golgi to the plasma membrane. Since there is no known experimental treatment to induce unstacking of Golgi cisternae in interphase cells, we exploited normal pathways that cause unstacking in mitosis. At the end of cell division, the Golgi membranes reassociate and reform stacks. In these experiments, we inhibited post-mitotic Golgi stack formation by microinjection of antibodies against GRASP65 or a dominant negative GRASP65 mutant protein. The observed cisternae were poorly aligned and only partially stacked (Fig. 1). The remaining partial stacking observed in our experiment may be due to the function of additional stacking factors or the cytoskeleton that concentrates the Golgi membrane in close proximity to the nucleus [11], [30].

Our results show that transport of CD8 from the ER through the Golgi to the cell surface more than doubled in cells with an unstacked Golgi, which is consistent with our results from the *in vitro* budding assay. The stimulation, however, may only be temporary. Initially, more vesicles are able to bud from the unstacked cisternae, translating into a higher rate of cargo transport. As more vesicles form, the rate of fusion could become a limiting factor that slows down vesicle transport. In this case, the stimulation of vesicle transport through the Golgi would be of a transient nature to induce a burst of membrane transport.

Mitotic fragmentation of the Golgi cisternae is regulated by cdk1/cyclin B1 [30], [31]. Phosphorylation of GM130 by this kinase abolishes the binding of p115 [17], and inhibits vesicle fusion. Continuous budding of vesicles leads to the disassembly of the Golgi [32]. However, this does not fully explain the vesiculation of the Golgi in prophase. Disruption of the p115 tether during interphase causes vesicle accumulation, but it takes over 60 min to convert 25% of cisternae into vesicles [22]. This is in contrast to mitosis, where the entire Golgi breaks down within minutes. Our results show that single cisternae vesiculate within 5–10 min, which is comparable to the Golgi disassembly between prophase and metaphase in cells [33]. This suggests that unstacking induced by GRASP65 phosphorylation plays a major role in the vesiculation of the Golgi in early mitosis and thereby accounts for the rapid disassembly of the Golgi. Unstacking of the cisternae increases the surface area where vesicles can form, resulting in the rapid breakdown of the Golgi during mitosis.

Our results provide the first evidence for the concept that the Golgi acts as a “flux regulator”. The speed of transport through the Golgi depends on the rate at which vesicles can form and fuse. In stacked cisternae, vesicles can bud and fuse only at the rims. This limits the amount of membrane where budding can occur and thus slows down the formation of vesicles and transport. Unstacking increases the surface area from which vesicles can form, resulting in an increase of protein delivery to the plasma membrane. The physiological relevance of stacked cisternae could be that cargo molecules are exposed to Golgi enzymes for a longer period, enhancing the chances of proper glycosylation and compensating for the relatively low activity of the glycosylation enzymes. An extended dwell time of the cargo could also compensate for the lack of a quality control mechanism in the Golgi for correct glycosylation patterns [34]. Using a mechanism like this, cells are able to actively regulate flux through the Golgi. Two interesting possibilities can be imagined. In situations where the correct pattern of glycosylation is more important, cells can slow down traffic of cargo molecules by enhancing stacking. In situations where transport speed and quantity are more desirable, the cells can actively increase the speed of transport by partially unstacking the Golgi.

## Materials and Methods

### Reagents

All reagents were from Sigma, Calbiochem or Roche Diagnostics unless otherwise stated. Purified antibodies against GRASP65 (residues 202–446) [12] were concentrated on a Microcon YM-10 filter (Millipore). Lysine-fixable Texas-Red and FITC 70 kDa dextrans and goat anti-mouse or goat anti-rabbit antibodies conjugated to Alexa Fluor 488 or Alexa Fluor 594 were from Invitrogen. The monoclonal antibody OKT8 against the luminal domain of CD8 (F. Watt, Cancer Research UK) was labeled with the Alexa Fluor 488 protein labeling kit (Invitrogen). The coupling ratio was 7.2 moles of dye per mole of antibody. Polyclonal antibodies against α-mannosidase II were from G. Warren. The monoclonal antibodies against GM130 and Gos28 were from Transduction Laboratories and secondary antibodies conjugated to HRP for Western blotting were from Jackson ImmunoResearch. The plasmid pcDNA CD8 was generated by subcloning the cDNA of CD8 from pCMUIV CD8 [19] into pcDNA3.1(+) (Invitrogen).

### Cell culture

Normal rat kidney (NRK) cells were grown at 37°C and 5% CO_2_ in DMEM, 100 U/ml penicillin, 100 µg/ml streptomycin (Invitrogen) and 10% fetal calf serum (Hyclone). For immuno-fluorescence and microinjections, cells were grown on glass coverslips or glass bottom dishes (MatTek).

### Recombinant proteins

His-tagged rat GRASP65 protein lacking the N-terminal myristoylation site (G2A GRASP65) was expressed in *E. coli* and purified on Ni-NTA agarose (Qiagen) [12]. Constitutively active human cdk1/cyclinB1 complexes were expressed in Hi5 insect cells and purified on Ni-NTA agarose [12]. Human polo-like kinase1 tagged with GST was expressed in *E. coli* and purified on Glutathione Sepharose 4B (GE Healthcare) [12], [35]. N-myristoylated ARF1 was expressed in *E. coli* and purified on a DEAE-Sepharose column (GE Healthcare) [18], [36].

### *In vitro* vesicle formation

Rat liver Golgi membranes [12] were treated with cdk1/cyclinB1 and plk1 in MEB buffer (50 mM Tris-HCl pH 7.3, 50 mM KCl, 10 mM MgCl_2_, 15 mM EGTA, 20 mM ß-glycerophosphate, 0.2 M sucrose, 2 mM ATP, 1 mM GTP, 1 mM glutathione) for 20 min at 37°C to induce cisternal unstacking. The membranes were re-isolated by pelleting (50,000 rpm in a TLA55 rotor for 20 min at 4°C) through a 0.4 M sucrose cushion in KHM buffer (25 mM HEPES pH 7.3, 60 mM KCl, 5 mM Mg(OAc)_2_, 0.2 M sucrose, 1 mM glutathione) and then treated with interphase HeLa cytosol [18] in KHM buffer with 10 µl anti-GRASP65 or G2A GRASP65 (5 mg/ml) for 60 min at 37°C. The membranes, or untreated Golgi stacks, were further treated with 10 µg coatomer [37], 5 µg ARF1, 1 mM GTP and an ATP regenerating system in KHM buffer. The reactions were fixed with 2% glutaraldehyde (EMS) in KHM buffer and prepared for EM [12]. The relative proportion of stacked and unstacked membranes and vesicles was determined by the intersection method [11]. The statistical significance was assessed by a two-tailed Student's *t* test.

Budding efficiency was also determined by separation of vesicles from the Golgi membranes followed by Western blotting according to an established protocol [38]. Briefly, after budding reactions were performed as described above, the samples were diluted with an equal volume of buffer that contained 500 mM KCl to release COPI vesicles. Samples were first centrifuged at 10,000 *g* for 15 min at 4°C in a swing out rotor to pellet the Golgi membranes. Vesicles in the supernatant were then sedimented through a layer of 0.5 ml 0.4 M sucrose in reaction buffer at 55000 rpm in a Beckman TLA55 rotor at 4°C. The pellets that contained Golgi membranes and vesicles were solubilized in SDS buffer and analyzed by SDS-PAGE and Western blotting. Results were quantified using the Image J software package.

### Microinjection, immunofluorescence and EM analyses

Microinjection was performed using a semi-automatic system from Eppendorf [22]. All microinjected proteins were in injection buffer. NRK cells were enriched in G1/S-phase with 2.5 µg/ml aphidicolin. The drug was removed after 5–6 h, and mitotic cells were injected with 7 mg/ml G2A GRASP65 together with 2 mg/ml rabbit IgGs (Sigma), 4 mg/ml anti-GRASP65, or 4 mg/ml rabbit IgGs as a control. 2 mg/ml FITC-dextran was co-injected as a marker. The cells were incubated for 3 h at 37°C followed by fluorescence or EM analysis.

For immunofluorescence studies, the cells were fixed for 15 min in 3.7% formaldehyde in PBS, permeabilized for 10 min in methanol at −20°C and labeled for GM130 (BD Biosciences) followed by goat anti-mouse Alexa Fluor594 antibodies. For EM, injected cells were identified by the FITC fluorescence of the co-injected dextran. Non-injected cells were scraped off the coverslip and the remaining injected cells that divided were processed for EM [22]. These cells were normally visible as pairs of daughter cells after cell division.

### Transport assay

To analyze the kinetics of CD8 transport during interphase, NRK cells were microinjected with 0.2 mg/ml pcDNA CD8 and 2 mg/ml Texas-Red dextran as a marker. Accumulation of CD8 on the cell surface was monitored by time-lapse microscopy as described below. To validate the assay, 5 mg/ml N73 peptide of GM130 (amino acids 1–73) was co-injected as an inhibitor of intra-Golgi transport [21], [22] or a control peptide from a non-Golgi protein UFD1 (amino acids 216–257). After 45 min at 37°C, the medium was changed to CO_2_-Independent Medium (Invitrogen), 10% fetal calf serum, 2 µg/ml OKT8-Alexa488 and 0.1 mg/ml cycloheximide. The cells were transferred to a Zeiss Axiovert 200 M microscope, which was enclosed (Incubator XL3, Zeiss) to maintain cells at 37°C. Injected cells were identified by the injected dextran and transport of CD8 was followed by the recruitment of fluorescence-labeled OKT8-Alexa488 to the cell surface. Images were captured at 2 min intervals with a LD Plan-Neofluar 40×/1.3 DIC or a Plan-Apochromat 63×/1.4 DIC objective (Zeiss) and an Orca-285 camera (Hamamatsu) using the software Openlab 4.02 (Improvision). The mean fluorescence intensity of the cells in each frame was quantified using Openlab [22]. The statistical significance was assessed by a two-tailed Student's *t* test.

## Supporting Information

Movie S1 Transport of CD8 to the cell surface, see Figure legend 2.
(0.92 MB MPG)

Movie S2 CD8 transport in post-mitotic cells with an unstacked Golgi, see Figure legend 3.
(0.15 MB MPG)

## References

[1] Marsh BJ, Volkmann N, McIntosh JR, Howell KE (2004). Direct continuities between cisternae at different levels of the Golgi complex in glucose-stimulated mouse islet beta cells.. Proc Natl Acad Sci U S A.

[2] Mironov AA, Beznoussenko GV, Polishchuk RS, Trucco A (2005). Intra-Golgi transport: a way to a new paradigm?. Biochim Biophys Acta.

[3] Trucco A, Polishchuk RS, Martella O, Di Pentima A, Fusella A (2004). Secretory traffic triggers the formation of tubular continuities across Golgi sub-compartments.. Nat Cell Biol.

[4] Elsner M, Hashimoto H, Nilsson T (2003). Cisternal maturation and vesicle transport: join the band wagon! (Review).. Mol Membr Biol.

[5] Losev E, Reinke CA, Jellen J, Strongin DE, Bevis BJ (2006). Golgi maturation visualized in living yeast.. Nature.

[6] Matsuura-Tokita K, Takeuchi M, Ichihara A, Mikuriya K, Nakano A (2006). Live imaging of yeast Golgi cisternal maturation.. Nature.

[7] Malhotra V, Mayor S (2006). Cell biology: the Golgi grows up.. Nature.

[8] Cai H, Reinisch K, Ferro-Novick S (2007). Coats, tethers, Rabs, and SNAREs work together to mediate the intracellular destination of a transport vesicle.. Dev Cell.

[9] Lupashin V, Sztul E (2005). Golgi tethering factors.. Biochim Biophys Acta.

[10] Pfeffer SR (2007). Unsolved mysteries in membrane traffic.. Annu Rev Biochem.

[11] Shorter J, Warren G (1999). A role for the vesicle tethering protein, p115, in the post-mitotic stacking of reassembling Golgi cisternae in a cell-free system.. J Cell Biol.

[12] Wang Y, Seemann J, Pypaert M, Shorter J, Warren G (2003). A direct role for GRASP65 as a mitotically regulated Golgi stacking factor.. Embo J.

[13] Barr FA, Puype M, Vandekerckhove J, Warren G (1997). GRASP65, a protein involved in the stacking of Golgi cisternae.. Cell.

[14] Colanzi A, Suetterlin C, Malhotra V (2003). Cell-cycle-specific Golgi fragmentation: how and why?. Curr Opin Cell Biol.

[15] Shorter J, Warren G (2002). Golgi architecture and inheritance.. Annu Rev Cell Dev Biol.

[16] Warren G (1993). Membrane partitioning during cell division.. Annu Rev Biochem.

[17] Lowe M, Rabouille C, Nakamura N, Watson R, Jackman M (1998). Cdc2 kinase directly phosphorylates the cis-Golgi matrix protein GM130 and is required for Golgi fragmentation in mitosis.. Cell.

[18] Xiang Y, Seemann J, Bisel B, Punthambaker S, Wang Y (2007). Active ARF1 is required for mitotic Golgi fragmentation.. J Biol Chem.

[19] Shima DT, Cabrera-Poch N, Pepperkok R, Warren G (1998). An ordered inheritance strategy for the Golgi apparatus: visualization of mitotic disassembly reveals a role for the mitotic spindle.. J Cell Biol.

[20] Wang Y, Satoh A, Warren G (2005). Mapping the functional domains of the Golgi stacking factor GRASP65.. J Biol Chem.

[21] Nakamura N, Lowe M, Levine TP, Rabouille C, Warren G (1997). The vesicle docking protein p115 binds GM130, a cis-Golgi matrix protein, in a mitotically regulated manner.. Cell.

[22] Seemann J, Jokitalo EJ, Warren G (2000). The role of the tethering proteins p115 and GM130 in transport through the Golgi apparatus in vivo.. Mol Biol Cell.

[23] Martinez-Menarguez JA, Prekeris R, Oorschot VM, Scheller R, Slot JW (2001). Peri-Golgi vesicles contain retrograde but not anterograde proteins consistent with the cisternal progression model of intra-Golgi transport.. J Cell Biol.

[24] Preisinger C, Körner R, Wind M, Lehmann WD, Kopajtich R (2005). Plk1 docking to GRASP65 phosphorylated by Cdk1 suggests a mechanism for Golgi checkpoint signalling.. EMBO J.

[25] Tang D, Mar K, Warren G, Wang Y (2007). Molecular mechanism of mitotic Golgi disassembly and reassembly revealed by a defined reconstitution assay.. J Biol Chem.

[26] Kondylis V, Spoorendonk KM, Rabouille C (2005). dGRASP localization and function in the early exocytic pathway in Drosophila S2 cells.. Mol Biol Cell.

[27] Sütterlin C, Polishchuk R, Pecot M, Malhotra V (2005). The Golgi-associated Protein GRASP65 Regulates Spindle Dynamics and Is Essential for Cell Division.. Mol Biol Cell.

[28] Puthenveedu MA, Bachert C, Puri S, Lanni F, Linstedt AD (2006). GM130 and GRASP65-dependent lateral cisternal fusion allows uniform Golgi-enzyme distribution.. Nat Cell Biol.

[29] Lane JD, Lucocq J, Pryde J, Barr FA, Woodman PG (2002). Caspase-mediated cleavage of the stacking protein GRASP65 is required for Golgi fragmentation during apoptosis.. J Cell Biol.

[30] Shorter J, Beard MB, Seemann J, Dirac-Svejstrup AB, Warren G (2002). Sequential tethering of Golgins and catalysis of SNAREpin assembly by the vesicle-tethering protein p115.. J Cell Biol.

[31] Lowe M, Barr FA (2007). Inheritance and biogenesis of organelles in the secretory pathway.. Nat Rev Mol Cell Biol.

[32] Rabouille C, Jokitalo E (2003). Golgi apparatus partitioning during cell division.. Mol Membr Biol.

[33] Zaal KJ, Smith CL, Polishchuk RS, Altan N, Cole NB (1999). Golgi membranes are absorbed into and reemerge from the ER during mitosis.. Cell.

[34] Helenius A, Aebi M (2001). Intracellular functions of N-linked glycans.. Science.

[35] Lin CY, Madsen ML, Yarm FR, Jang YJ, Liu X (2000). Peripheral Golgi protein GRASP65 is a target of mitotic polo-like kinase (Plk) and Cdc2.. Proc Natl Acad Sci U S A.

[36] Franco M, Chardin P, Chabre M, Paris S (1995). Myristoylation of ADP-ribosylation factor 1 facilitates nucleotide exchange at physiological Mg2+ levels.. J Biol Chem.

[37] Pavel J, Harter C, Weiland FT (1998). Reversible dissociation of coatomer - functional-characterization of a beta/delta-coat protein subcomplex.. Proc Natl Acad Sci U S A.

[38] Sonnichsen B, Lowe M, Levine T, Jamsa E, Dirac-Svejstrup B (1998). A role for giantin in docking COPI vesicles to Golgi membranes.. J Cell Biol.

